# THE IMPLEMENTATION PROCESS OF AN INTERGENERATIONAL COMMUNITY RESEARCH PROJECT: A NARRATIVE REVIEW

**DOI:** 10.1093/geroni/igac059.1055

**Published:** 2022-12-20

**Authors:** Rachel Scrivano, Jill Juris, Shannon Jarrott

**Affiliations:** The Ohio State University, Columbus, Ohio, United States; Appalachian State University, Boone, North Carolina, United States; The Ohio State University, Columbus, Ohio, United States

## Abstract

Food for a Long Life (FFLL) was a five-year USDA Children, Youth and Families at Risk intergenerational community research project that sought to increase healthy food consumption, knowledge, and access among preschoolers and older adults living in food insecure communities of Ohio and Virginia. Using the community-based participatory action research approach, community stakeholders jointly participated in all stages of the project to co-create context-specific programming to address needs within their communities with the goal of promoting program sustainability beyond the grant funding period. This presentation will provide a narrative review to explore the implementation process of FFLL by comparing initial project expectations to reality using the community-based participatory research conceptual model (Wallerstein et al., 2008; Wallerstein et al., 2018). By exploring project barriers such as COVID-19, facilitators such as flexible partner relationships, and strategies including promoting early buy-in, we provide an in-depth discussion of project successes and lessons learned.

